# EWSR1-WT1 Target Genes and Therapeutic Options Identified in a Novel DSRCT In Vitro Model

**DOI:** 10.3390/cancers13236072

**Published:** 2021-12-02

**Authors:** Margit Bleijs, Corine Pleijte, Sem Engels, Femke Ringnalda, Friederike Meyer-Wentrup, Marc van de Wetering, Hans Clevers

**Affiliations:** 1 Princess Máxima Center for Pediatric Oncology, 3584 CT Utrecht, The Netherlands; m.w.bleijs-2@prinsesmaximacentrum.nl (M.B.); cpleijte@umcutrecht.nl (C.P.); s.a.g.engels-3@prinsesmaximacentrum.nl (S.E.); f.c.a.ringnalda@prinsesmaximacentrum.nl (F.R.); f.meyer-wentrup@prinsesmaximacentrum.nl (F.M.-W.); m.l.vandewetering@prinsesmaximacentrum.nl (M.v.d.W.); 2 Oncode Institute, 3521 AL Utrecht, The Netherlands; 3 Hubrecht Institute, Royal Netherlands Academy of Arts and Sciences and University Medical Center, 3584 CT Utrecht, The Netherlands

**Keywords:** DSRCT, EWSR1-WT1, preclinical model, in vitro model, pediatric cancer, sarcoma

## Abstract

**Simple Summary:**

Desmoplastic small round cell tumor (DSRCT) is an extremely rare soft tissue sarcoma arising in the abdomen of adolescents and young adults. This sarcoma is driven by a single genomic rearrangement, resulting in the expression of the *EWSR1-WT1* fusion gene. No effective treatment exists for DSRCT patients, which highlights the need for preclinical models to study disease progression and drug sensitivity. The aim of this study is to develop a pre-clinical DSRCT in vitro model, which enables investigating the molecular target genes of the *EWSR1-WT1* fusion gene and allows for medium-throughput drug screening to discover new therapeutic options.

**Abstract:**

Desmoplastic small round cell tumor (DSRCT) is a rare and aggressive soft tissue sarcoma with a lack of effective treatment options and a poor prognosis. DSRCT is characterized by a chromosomal translocation, resulting in the *EWSR1-WT1* gene fusion. The molecular mechanisms driving DSRCT are poorly understood, and a paucity of preclinical models hampers DSRCT research. Here, we establish a novel primary patient-derived DSRCT in vitro model, recapitulating the original tumor. We find that *EWSR1-WT1* expression affects cell shape and cell survival, and we identify downstream target genes of the EWSR1-WT1 fusion. Additionally, this preclinical in vitro model allows for medium-throughput drug screening. We discover sensitivity to several drugs, including compounds targeting RTKs. MERTK, which has been described as a therapeutic target for several malignancies, correlates with *EWSR1-WT1* expression. Inhibition of MERTK with the small-molecule inhibitor UNC2025 results in reduced proliferation of DSRCT cells in vitro, suggesting MERTK as a therapeutic target in DSRCT. This study underscores the usefulness of preclinical in vitro models for studying molecular mechanisms and potential therapeutic options.

## 1. Introduction

Desmoplastic small round cell tumor (DSRCT) is a highly aggressive and rare soft tissue sarcoma [1,2,3]. This sarcoma was first characterized as a separate entity by Gerald and Rosai in 1989, who described the histologic appearance of DSRCT as nests of small round blue cells, separated by desmoplastic stroma [4]. DSRCT exhibits features of multi-phenotypic differentiation, since mesenchymal, epithelial, and neural markers are expressed within this sarcoma [3,4]. Genetically, DSRCT is characterized by a chromosomal translocation, resulting in a gene fusion involving Ewing sarcoma region 1 (*EWSR1*) and Wilms tumor 1 (*WT1*). The chimeric protein contains the N-terminal domain of EWSR1 fused to the DNA-binding domain of WT1, including zinc fingers 2–4 [5]. The N-terminal domain of EWSR1 is thought to act as a transcriptional activator, while the zinc finger domains of WT1 bind DNA of regulatory elements in its target genes [6]. Previously, it has been shown that two different isoforms of *EWSR1-WT1* are expressed, distinguished by the presence or absence of a three amino acid KTS domain, which induce distinct transcriptional profiles [5,7,8].

Recently, a very comprehensive study classified molecular subtypes of sarcomas, including DSRCT [9]. Several downstream targets of its *EWSR1-WT1* fusion gene were identified. Kang and colleagues showed that EWSR1-WT1 directly binds to the promoter of neural reprogramming factor *ASCL1*, activating neural gene expression [7]. Gedminas and colleagues identified EWSR1-WT1 target genes using an siRNA knock-down model of the fusion in the patient-derived JN-DSRCT-1 and patient-derived xenograft (PDX)-derived BER cell lines [10,11,12]. They discovered several downstream target pathways that are commonly deregulated in fusion-positive sarcomas and observed an overlap between EWSR1-WT1 and EWSR1-FLI1 (the fusion gene driving Ewing sarcoma) gene signatures. Hingorani and colleagues performed bulk RNA sequencing of patient-derived DSRCT specimens and identified CD200 and CD276 as potentially targetable immune checkpoint molecules, whose expression are independent of *EWSR1-WT1* expression. Additionally, they performed WT1 ChIP-sequencing and established an shRNA knock-down model of *EWSR1-WT1* in the JN-DSRCT-1 cell line and identified IGF2 and FGFR4 as potential therapeutic targets in DSRCT patients [13]. While these studies give important insights on the downstream targets of the *EWSR1-WT1* fusion gene, molecular mechanisms triggered by the gene fusion, which account for the aggressive proliferation of DSRCT, have remain elusive.

Preclinical models, such as cell lines, organoids, and patient-derived xenografts (PDXs), are key for investigating tumor progression and molecular mechanisms [14]. While several PDX models have been established from DSRCT specimens by transplantation into the flank of the mouse [11], only two in vitro models of DSRCT have been published: one primary patient-derived and one PDX-derived cell line [10,11,12]. The paucity of preclinical DSRCT models hampers the research on mechanisms driving DSRCT and discovery of therapeutic options. Here, we establish a novel primary patient-derived DSRCT model in vitro, which recapitulates morphological and transcriptomic features of the originating tumor. We use a shRNA knock-down model to identify molecular target genes of the EWSR1-WT1 fusion. Additionally, this preclinical in vitro model allows for medium-throughput drug screening to discover drug sensitivity in DSRCT cells. Finally, we discover promising therapeutic targets, including an EWSR1-WT1-driven receptor tyrosine kinase (RTK).

## 2. Materials and Methods

### 2.1. Patient-Derived DSRCT Specimen

Surgically resected tissue was obtained from a DSRCT patient at the Amsterdam UMC with informed consent. Tumor material was washed with Advanced Dulbecco’s Modified Eagle’s Medium F12 (AdDMEM-F12) (Gibco™, Thermo Fisher Scientific, #12634010, Waltham, MA, USA), supplemented with 1% pen/strep (Gibco™, Thermo Fisher Scientific, #15140122, Life Technologies, Merelbeke, Belgium), 1% Glutamax (Gibco™, Thermo Fisher Scientific, #35050038, Life Technologies, Merelbeke, Belgium), 1% Hepes (Gibco™, Thermo Fisher Scientific, #15630056, Life Technologies, Merelbeke, Belgium), and minced into tumor pieces using scalpels, as well as mechanically disrupted by pipetting up and down before plating into suspension cell culture plates.

### 2.2. Cell Culture

The patient-derived OV-054 DSRCT cell line was maintained in DSRCT medium, containing Advanced Dulbecco’s Modified Eagle’s Medium F12 (AdDMEM-F12) (Gibco™, Thermo Fisher Scientific, #12634010, Waltham, MA, USA), supplemented with 1% pen/strep (Gibco™, Thermo Fisher Scientific, #15140122, Life Technologies, Merelbeke, Belgium), 1% Glutamax (Thermo Fisher Scientific, #35050038), 1% N2 (Gibco, #17502048), 1% Hepes (Gibco™, Thermo Fisher Scientific, #15630056, Life Technologies, Merelbeke, Belgium), 2% B27 supplement minus vitamin A, 50x (Gibco™, Thermo Fisher Scientific, #12587010, Life Technologies, Merelbeke, Belgium), 0.25% N-Acetyl-L-cysteine (NAC) (Sigma Aldrich, #A9165, St. Louis, MO, USA), 50 ng/mL FGF-basic (154 a.a.) (PeproTech, #100-18B, Rocky Hill, NJ, USA), 50 ng/mL EGF (PeproTech, #AF-100-15, Rocky Hill, NJ, USA), 10 ng/mL IGF1 (PeproTech, #100-11, Rocky Hill, NJ, USA), 10 ng/mL Rho Kinase Inhibitor (AbMole BioScience, #M1817, Houston, TX, USA), 10 ng/mL BMP4 (PeproTech, #120-05ET, Rocky Hill, NJ, USA), and 0.1% BME (Cultrex Reduced Growth Factor Basement Membrane Extract, Type 2, #3533-005-2, Bio-Techne, Minneapolis, MN, USA). Cells were incubated at 37 °C with 5% CO_2_ and passaged 1:5 every 10 days using TrypLE™ Express Enzyme (Gibco™, Thermo Fisher Scientific, #12605010, Life Technologies, Merelbeke, Belgium).

HEK293T cells were maintained in Dulbecco’s Modified Eagle’s Medium (Gibco™, Thermo Fisher Scientific, #31966-047, Life Technologies, Merelbeke, Belgium), supplemented with 10% Fetal Calf Serum (FCS) (Gibco™, Thermo Fisher Scientific, #A4766801, Life Technologies, Merelbeke, Belgium), 1% pen/strep (Gibco™, Thermo Fisher Scientific, #15140122, Life Technologies, Merelbeke, Belgium), and 1% Hepes (Gibco™, Thermo Fisher Scientific, #15630056, Life Technologies, Merelbeke, Belgium). Cells were incubated at 37 °C with 5% CO_2_ and passaged 1:20 every 3–4 days using TrypLE™ Express Enzyme (Gibco™, Thermo Fisher Scientific, #12605010, Life Technologies, Merelbeke, Belgium).

JN-DSRCT-1 cells were maintained in Dulbecco’s Modified Eagle’s Medium F12 (DMEM-F12) (Gibco™, Thermo Fisher Scientific, #11320033, Waltham, MA, USA), supplemented with 1% pen/strep (Gibco™, Thermo Fisher Scientific, #15140122, Life Technologies, Merelbeke, Belgium) and 10% Fetal Calf Serum (FCS) (Gibco™, Thermo Fisher Scientific, #A4766801, Life Technologies, Merelbeke, Belgium). Cells were incubated at 37 °C with 5% CO_2_ and passaged 1:10 every 3–4 days using TrypLE™ Express Enzyme (Gibco™, Thermo Fisher Scientific, #12605010, Life Technologies, Merelbeke, Belgium).

### 2.3. Single-Cell RNA Sequencing

OV-054 DSRCT cells were digested using TrypLE™ Express Enzyme (Gibco™, Thermo Fisher Scientific, #12605010, Life Technologies, Merelbeke, Belgium) and mechanically dissociated by pipetting up and down. The 2D and 3D OV-054 DSRCT cells were subsequently sorted for DAPI negative cells using the FACSJazz (BD bioscience, Franklin Lakes, NJ, USA) and SH800S Cell Sorter (SONY Europe B.V., Weybridge, Surrey, UK), respectively. Single DSRCT cells were sorted into a 384-well plate for SORT-seq performed by Single Cell Discoveries B.V. [15]. Gene counts per gene per well are provided in Tables S1–S4. Cluster analysis was performed using RaceID3 [16]. WT1 reads represent *EWSR1-WT1* fusion expression, since wild-type WT1 is not expressed in OV-054 DSRCT cells.

### 2.4. Comparison R2 Dataset

The DSRCT patient described in this paper is included in the INFORM study [17] and available microarray data were analyzed in R2 (www.r2.amc.nl, accessed on 14 January 2020). Two gene sets were created from the gene expression list of the original DSRCT patient: high-expressed genes (Z-score > 1) and low-expressed genes (Z-score < −1). Average read counts of the genes in these gene sets were collected from the here generated scRNAseq data of the 2D and 3D cultured DSRCT cells.

### 2.5. Introduction of shRNAs into OV-054 DSRCT Cells

shRNAs targeting the *EWSR1-WT1* breakpoint or *WT1* exon 8–10 and a non-targeting shRNA were cloned into pLKO-Tet-On Vector (Addgene, #21915) (Table S5). shRNAs were introduced in the patient-derived OV-054 DSRCT cells using lentiviral transduction. Cells were selected by addition of 0.5 µg/mL puromycin (InvivoGen, #ant-pr-1, San Diego, CA, USA) to the culture medium. Transcription of shRNAs was induced with 1.0 μg/mL doxycycline hyclate (Sigma Aldrich, #D9891, St. Louis, MO, USA). For shRNA 3, we harvested RNA 8, 24, and 32 h post-doxicyclin induction. For shRNA 2, we harvested RNA 40 h post-doxicyclin induction.

### 2.6. RNA Isolation, PCR, and qPCR

RNA isolation was performed using Direct-zol RNA MicroPrep Kit (Zymo Research, R2062, Orange, CA, USA), according to the manufacturer’s protocol. cDNA synthesis was performed using SuperScript III reverse transcriptase (Thermo Fisher Scientific, #12574026, Life Technologies, Bleiswijk, The Netherlands) and random primers (Promega, #C1181, Madison, WI, USA). q-PCR was performed using iQ^TM^ SYBR Green Supermix (BIO-RAD Laboratories, #1708882, Hercules, CA, USA). *EWSR1-WT1* was amplified from cDNA by the primers FW: 3′-TCCTACAGCCAAGCTCAAGTC-5′ and REV: 3′-ACCTTCGTTCACAGTGGTTG-5′. *MERTK* was amplified from cDNA by the primers FW: 3′-GTGTCCAAGGGAGTGCAG-5′ and REV: 3′-CTCAGCGGATCAGCTTCC-5′. Cq-values were normalized to *GAPDH* and amplified by the primers: FW: 3′-CACATCGCTCAGACACCATG-5′ and REV: 3′-TGACGGTGCCATGGAATTTG-5′. For PCR and sequencing of the *EWSR1-WT1* breakpoint, the same primers were used, as mentioned above, for the q-PCR. The reverse primer was used for sequencing of the fusion breakpoint by Macrogen Europe BV.

### 2.7. RNA Sequencing

RNA samples of OV-054 DSRCT cells, upon induction of shRNA 2, 3, and NT, were paired-end sequenced by Macrogen Europe BV, using Illumina TruSeq stranded total RNA library construction with Ribo-Zero Gold and Novaseq6000 S4 2 × 150 bp. To allow for distinguishing between reads that derived from *EWSR1-WT1* fusion transcripts, wild-type *EWSR1* transcripts, or wild-type *WT1* transcripts, the hg38 reference transcriptome was obtained from Ensembl and edited. Therefore, the full-length *EWSR1* was split into 5′*EWSR1* (exon 1–7) and 3′*EWSR1* (exon 8–17) and full-length *WT1* was split into 5′*WT1* (exon 1–7) and 3′*WT1* (exon 8–10). Paired-end sequencing reads were aligned to this edited reference transcriptome using the Burrows–Wheeler aligner software package (BWA-0.7.17) [18]. RPKM-normalized read counts are provided in Tables S6 and S7. Transcript count analysis and figures were made using R (http://www.r-project.org, accessed on 30 April 2020).

### 2.8. Time-Lapse Imaging and Particle Analysis

OV-054 DSRCT cells with shRNA 3 and NT shRNA were induced with DOX, 24 h prior to live imaging. Live imaging was performed using the Leica DMi8, while incubated at 37 °C and 5% CO_2_. Images were taken every 2 min from representative areas in each well for 16 h in total (24–40 h post-DOX). Cell counts and cell area from pictures of DOX-induced and non-induced shRNAs in OV-054 DSRCT cells were analyzed using particle analysis in ImageJ (National Institutes of Health and the Laboratory for Optical and Comutational Instrumentation (LOCI, University of Wisconsin), Madison, WI, USA). The number of segments represent the number of cells, and the segment areas represent the area of each cell. Cell migration and cell adhesion genes were identified using the DAVID gene annotation tool [19,20]. Cell-matrix interactions were quantified using the cell counter tool in ImageJ (National Institutes of Health and the Laboratory for Optical and Comutational Instrumentation (LOCI, University of Wisconsin), Madison, WI, USA).

### 2.9. Medium-Throughput Compound Screen

Two days prior to the addition of compounds, 5 × 10^3^ OV-054 DSRCT cells were seeded, per well, in 384-well plates. A panel of 201 drugs and DMSO controls were added to the 384-well plates in different concentrations using the Beckman Coulter Biomek i7 Hybrid liquid handling workstation. After 5 days of exposure of the compounds, cell viability was measured with CellTiter-Glo^®^ assay (Promega, #G9681, Madison, WI, USA) using the Spectramax i3x luminescence microplate reader. Values were normalized to DMSO. AUC Z-scores were calculated by (AUC-AUC_avg_)/st.dev for OV-054 DSRCT cultures and two Ewing cultures (ES-046 and ES-041). Relevant plasma concentrations from previous phase I and II trials (Table S8) were plotted to compare the viability curves.

### 2.10. UNC2025 Screen

OV-054 DSRCT cells, JN-DSRCT-1 cells, and control human small intestinal (huSI) organoids were incubated with different concentrations of MERTK inhibitor UNC2025 (Selleckchem, #S7576, Houston, TX, USA): 25, 50, 100, 200, or 400 nM was added to the culture medium. Seven days after the addition of UNC2025, representative pictures were taken, and cells were treated with TryplE™ Express Enzyme (Gibco™, Thermo Fisher Scientific, #12605010, Life Technologies, Merelbeke, Belgium) and counted. Viability of huSI organoids were measured by organoid size using representative pictures and the area measurement tool in ImageJ (National Institutes of Health and the Laboratory for Optical and Comutational Instrumentation (LOCI, University of Wisconsin), Madison, WI, USA).

## 3. Results

### 3.1. Establishment and Characterization of a DSRCT In Vitro Preclinical Model

**Figure 1:**
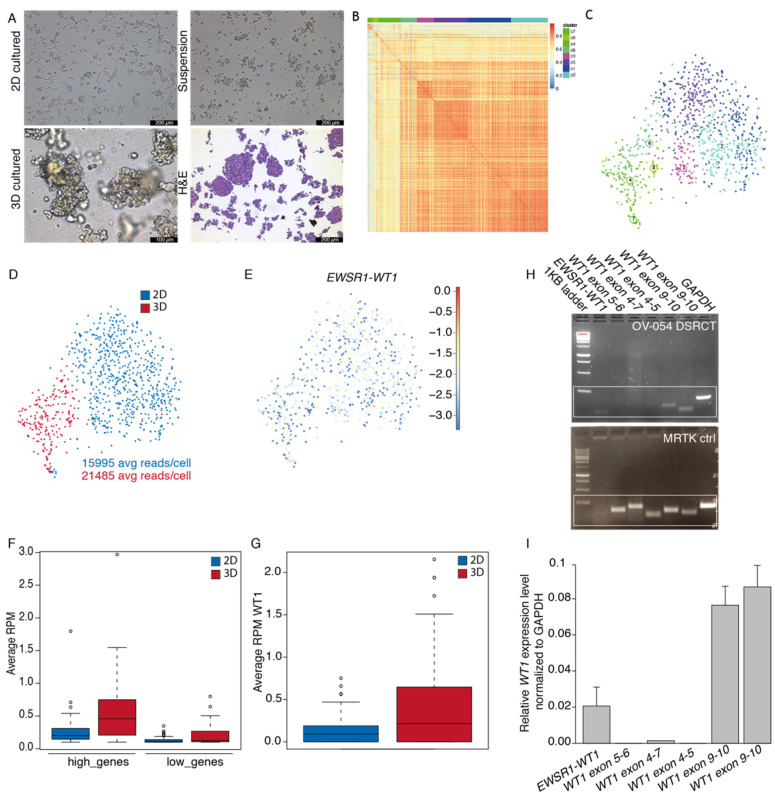
Establishment of a patient-derived DSRCT model in vitro. (**A**) Pictures of OV-054 DSRCT cells in different growth conditions: 2D on 0.1% BME, 3D in BME droplets and in suspension without BME. H&E staining is performed on cells in suspension. Scale bar: 100 µm (3D) and 200 µm (2D, suspension, and H&E). (**B**) Heatmap depicting cell clustering, based on differentially expressed genes. (**C**) tSNE plot depicting clusters of OV-054 DSRCT cells. (**D**) tSNE plot depicting clustering of 2D (blue) and 3D (red) cultured OV-054 DSRCT cells. (**E**) tSNE plot depicting *EWSR1-WT1* expression in 2D and 3D OV-054 DSRCT cells. (**F**) Box plot depicting the average RPM in 2D (blue) and 3D (red) cultured DSCRT cells for highly expressed and lowly expressed gene sets. (**G**) Box plot depicting the average *WT1* RPM in 2D (blue) and 3D (red) cultured OV-054 DSRCT cells. (**H**) Picture depicting gel electrophoresis of PCR amplicons of the *EWSR1-WT1* fusion breakpoint and different regions of *WT1* from cDNA of OV-054 DSRCT cells and MRTK control cells. (**I**) Bar plot depicting relative expression of *EWSR1-WT1* and different regions of *WT1* by qPCR, normalized to *GAPDH*.

Surgically resected tissue was obtained from a DSRCT patient and successfully cultured in vitro under various conditions, including as spheroids in 3D basement membrane extract type 2 (BME) droplets, as a 2D layer in 0.1% BME, and in suspension without BME (Figure 1A). The DSRCT in vitro cultures were established and maintained for over two years in serum-free basal medium (AdDMEM/F12+), supplemented with the specific growth factors EGF, FGF2, IGF1, RKI, and BMP4. While these growth factors were essential for initial growth of DSRCT cells, the established OV-054 DSRCT cultures were able to be maintained without the additional growth factors EGF, FGF2, IGF1, RKI, and BMP4 for over 10 passages. The addition of the growth factors independently did not affect DSRCT cell expansion in vitro, compared to the control medium without the additional growth factors (Figure S1), suggesting that these OV-054 DSRCT cells are able to adapt fast and are able to propagate independently of their microenvironment. For consistency, all further experiments were conducted using the complete medium, in which the OV-054 DSRCT model was established. H&E staining on OV-054 DSRCT cultures revealed nests of small round cells with large nuclei (Figure 1A), which is typically seen in DSRCT tissue [4]. Generally, patient-derived tumor tissue grown in 3D BME droplets can form organized structures, so-called organoids, that can capture disease heterogeneity [21]. To investigate whether our 2D and 3D DSRCT in vitro models also consist of distinct cell populations, single-cell RNA sequencing (scRNAseq) was performed. scRNAseq analysis of 2D and 3D OV-054 DSRCT cultures revealed a homogeneous, tight population of DSRCT cells (Figure 1B,C). The slight difference between clustering of 2D versus 3D cultured cells was likely due to sequencing depth variation (Figure 1D). Approximately 0–2 reads, per million (RPM) of *WT1*, were picked up per cell in the scRNAseq analysis (Figure 1G), indicating low *EWSR1-WT1* expression. Importantly, the expression of *EWSR1-WT1* was similar between the 2D and 3D cultures (Figure 1E,G).

The DSRCT patient, described in this study, was enrolled in the INFORM (individualized therapy for relapsed malignancies in childhood) pilot study [17]. The INFORM registry applied comprehensive molecular profiling to provide information on actionable gene variants, which may be used for subsequent therapeutic approaches. Microarray data of the original tumor of this DSRCT patient were obtained from the INFORM study and used to create two gene sets from the gene expression list: high (Z-score > 1) and low genes (Z-score < −1). Average read counts of the two gene sets were collected from the scRNAseq data of the 2D and 3D cultured DSRCT cells, and a similar trend was found between expression of the gene sets in the in vitro cultures, compared to the original tumor (Figure 1F). This indicated that both the 2D and 3D cultured DSRCT cells closely resemble features of the original tumor. For consistency, subsequent experiments were conducted with 2D cultured OV-054 DSRCT cells. To check whether wild-type *WT1* is expressed in OV-054 DSRCT cells, the *EWSR1-WT1* fusion breakpoint from cDNA, as well as several regions of *WT1*, were amplified. In our in vitro OV-054 DSRCT model, only the 3′-most exons of *WT1* were expressed (Figure 1H,I), while the expression of *WT1* exon 4–7 was absent. This indicated that exons 9–10 from *WT1* were derived from the *EWSR1-WT1* fusion cDNA and that wild-type *WT1* was not expressed in these cells (Figure 1H,I). As a control, cDNA of a *WT1*-expressing malignant rhabdoid tumor of the kidney (MRTK) model was taken along, which, indeed, showed expression of all regions of the *WT1* gene (Figure 1H).

### 3.2. shRNAs Targeting the EWSR1-WT Breakpoint Create an Effective Knock-Down of the Fusion mRNA

**Figure 2:**
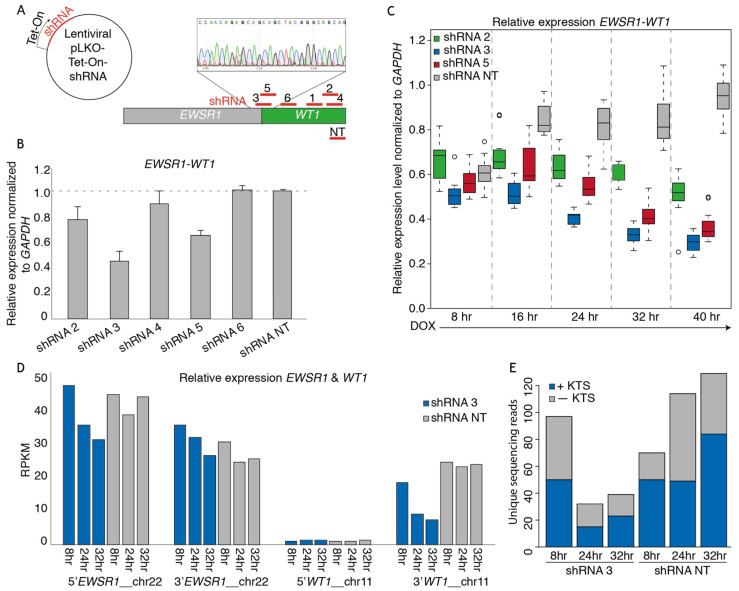
shRNA-mediated *EWSR1-WT1* knock-down in the DSRCT in vitro model. (**A**) Animation depicting the design of shRNAs targeting the breakpoint of *EWSR1-WT1*, 3′*WT1*, and a non-targeting (NT) shRNA. shRNAs were cloned into a lentiviral Tet-On backbone. (**B**) Bar plot depicting relative *EWSR1-WT1* expression levels 24 h post-DOX-induction of shRNAs normalized to *GAPDH*, as determined by qPCR. (**C**) Box plot depicting relative *EWSR1-WT1* expression levels at 8, 16, 24, 32, and 40 h post-DOX-induction of shRNA 2, 3, 5, and NT normalized to *GAPDH*, as determined by qPCR. (**D**) Bar plot depicting RPKM counts of 5′*EWSR1*, 3′*EWSR1*, 5′*WT1*, and 3′*WT1* at 8, 24, and 32 h post-DOX-induction of shRNA 3 and NT, as determined by mRNA paired-end sequencing. (**E**) Bar plot depicting number of +KTS (blue) and −KTS (gray) isoform reads, as determined by mRNA paired-end sequencing.

The OV-054 DSRCT in vitro model enabled us to study the molecular target genes of the fusion driving DSRCT. By generating a knock-down model of the *EWSR1-WT1* fusion mRNA, target genes could be identified that were affected by the expression level of *EWSR1-WT1*. To this end, we sequenced the breakpoint of *EWSR1-WT1* cDNA, in order to identify the exact location of the gene-fusion and found that *EWSR1* exon 7 was fused to *WT1* exon 8. This enabled us to design short hairpin RNAs (shRNAs), targeting the exact *EWSR1-WT1* breakpoint (shRNA 3, 5) or exon 8–10 of *WT1* (shRNA 2, 4, 6). With these, we created a DOX-inducible knock-down of the *EWSR1-WT1* fusion mRNA (Figure 2A). As a control, a non-targeting (NT), DOX-inducible scrambled shRNA sequence was taken along. An effective knock-down of the *EWSR1-WT1* fusion was observed with shRNA 2, 3, and 5 after 24 h of DOX induction (Figure 2B). When cells were harvested 8, 16, 24, 32, and 40 h after DOX induction, a gradual decrease of *EWSR1-WT1* transcripts was observed (Figure 2C), where shRNA 3 resulted in the most efficient knock-down. To identify genes that are affected upon *EWSR1-WT1* knock-down, RNA sequencing was performed. RNA sequencing reads were mapped to a reference transcriptome, in which *EWSR1* and *WT1* were split into a 3′end and a 5′end, in order to distinguish between the *EWSR1-WT1* fusion and wild-type *EWSR1* and *WT1* transcripts. 5′*WT1* was not expressed (Figure 2D), again confirming that wild-type *WT1* was not expressed in these DSRCT cells; 5′*EWSR1* and 3′*WT1* showed a gradual decrease upon DOX induction (Figure 2D), indicative of efficient knock-down of the *EWSR1-WT1* fusion transcripts. A slight decrease was seen in 3′*EWSR1* transcripts (Figure 2D), suggesting that shRNA 3 could also target wild-type *EWSR1*. However, the RPKM did not drop below the 3′*EWSR1* levels in the NT shRNA control, indicating that this effect was minimal. Sequencing reads were picked-up from both +KTS and −KTS isoforms, which were decreased to a similar extent after induction of shRNA 3 (Figure 2E). Hence, knock-down of the *EWSR1-WT1* fusion with shRNA 3 did not result in an isoform bias. Together, these data showed that induction of shRNA 3 (targeting the *EWSR1-WT1* breakpoint in DSRCT cells) resulted in an efficient knock-down of the fusion gene. Wild-type *EWSR1* and *WT1* were not affected, and no bias was observed for the +KTS or −KTS isoform. This *EWSR1-WT1* knock-down model was used to further investigate genes affected by levels of *EWSR1-WT1* expression.

### 3.3. EWSR1-WT1 Expression Affects Cell Shape, Cell Propagation, and the Transcriptome

**Figure 3:**
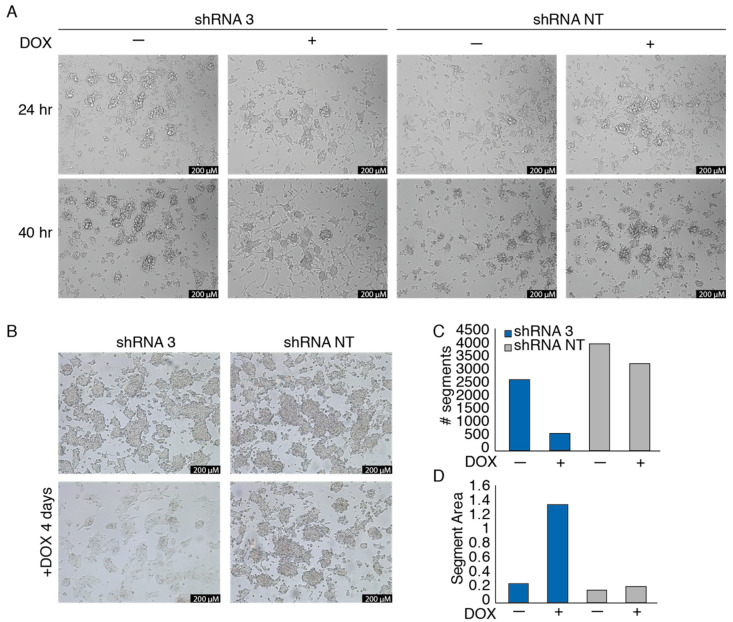
*EWSR1-WT1* expression affects OV-054 DSRCT cell shape and propagation. (**A**) Time-lapse images of OV-054 DSRCT cells upon *EWSR1-WT1* knock-down 24 and 40 h post-DOX-induction; scale bar: 200 µm. (**B**) Representative pictures taken 4 days post-DOX-induction of shRNA 3 and shRNA NT in DSRCT cells; scale bar: 200 µm. (**C**) Bar plot depicting number of segments, representing OV-054 DSRCT cells 4 days post-DOX-induction of shRNA 3 and shRNA NT, using particle analysis in ImageJ. (**D**) Bar plot depicting segment area, representing OV-054 DSRCT cells found 4 days post-DOX-induction of shRNA 3 and shRNA NT, using particle analysis in ImageJ.

To investigate the phenotypic effects of EWSR1-WT1 in OV-054 DSRCT cells, time-lapse imaging was performed. Knock-down of the *EWSR1-WT1* fusion mRNA, in a time lapse between 24 h and 40 h after DOX induction, resulted in a decrease of cell–cell adhesion and increase of cell-matrix adhesion (Figure 3A and Figure S2). Additionally, after 4 days of DOX-induction of shRNA 3, OV-054 DSRCT cells appeared more stretched, creating more cell-matrix adhesion, while non-induced cells, as well as cells with DOX-induction of the NT shRNA control, remained in clusters of inter-adhesive cells (Figure 3B). Upon knock-down of *EWSR1-WT1*, the number of cells decreased (Figure 3C), while the area of the cells increased (Figure 3D), showing that decreased expression of *EWSR1-WT1* quickly affects shape and expansion of DSRCT cells. 

**Figure 4:**
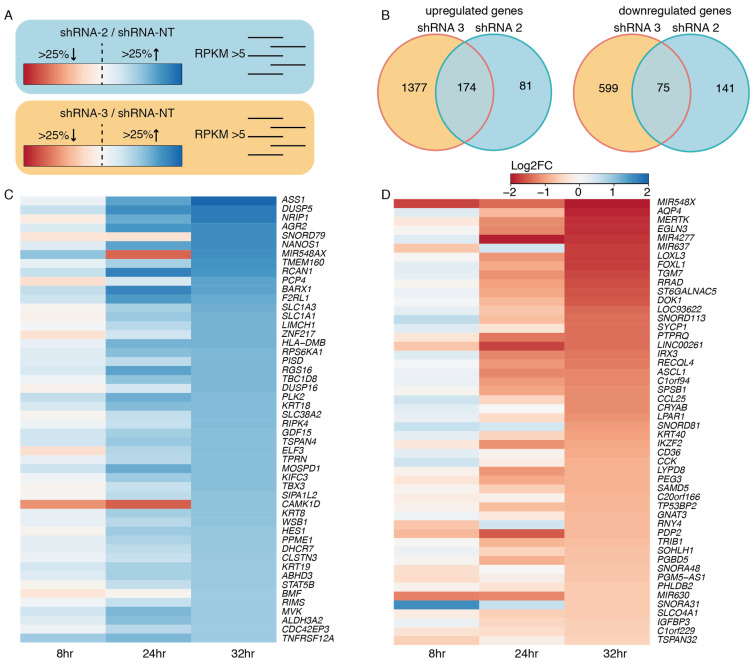
Target genes of EWSR1-WT1. (**A**) Animation of the approach to find EWSR1-WT1 target genes: select genes that are at least 25% up- or downregulated and have an RPKM value >5 for both shRNA 2 and shRNA 3. (**B**) Venn diagram depicting upregulated and downregulated genes, upon knock-down of *EWSR1-WT1* with shRNA 2 and shRNA 3. (**C**) Heatmap depicting the 50 highest upregulated genes, upon *EWSR1-WT1* knock-down, colors represent log2FC, compared to shRNA NT. (**D**) Heatmap depicting 50 most downregulated genes, upon *EWSR1-WT1* knock-down, colors represent log2FC, compared to shRNA NT.

To investigate the effect of *EWSR1-WT1* expression on the transcriptome, RNA sequencing was performed 8, 24, and 32 h after induction of shRNA 2, 3, and NT. To control for off-target effects, both shRNA 2 and shRNA 3 were used, which target *WT1* exon 10 and the *EWSR1-WT1* breakpoint, respectively. For both shRNA 2 and shRNA 3, genes that were at least 25% up- or downregulated were selected, compared to the NT shRNA control. A cut-off was set at 5 RPKM, in order to extract truly expressed genes. Genes that were affected by both shRNA 2- and shRNA 3-mediated knock-down of the *EWSR1-WT1* fusion were called true target genes of EWSR1-WT1 (Figure 4A). After 32 h of DOX-induction, we identified 75 genes that were downregulated upon *EWSR1-WT1* knock-down and 174 genes that were upregulated, upon *EWSR1-WT1* knock-down (Figure 4B). The expression levels of the 50 most upregulated genes and most downregulated genes, upon *EWSR1-WT1* knock-down, are shown in heatmaps (Figure 4C,D). The phenotypic effects that we observed were accompanied by an increased expression of several cell migration and cell adhesion genes (Figure S3C). While phenotypically the DSRCT cells appeared more mesenchymal upon knock-down of the *EWSR1-WT1* fusion, no evidence was found for epithelial to mesenchymal transition (EMT) (Figure S3D) [22]. Together, these data showed that cell migration and adhesion genes are upregulated upon knock-down of *EWSR1-WT1*, resulting in phenotypic changes, including a decreased cell expansion and increase in cell surface area. Subsequently, OV-054 DSRCT cells, with decreased *EWSR1-WT1* levels, create more cell-matrix interactions (Figure S3A,B).

**Figure 5:**
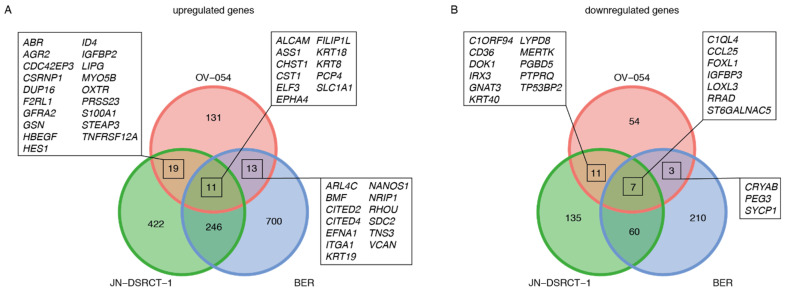
Comparison of *EWSR1-WT1* target genes in OV-054, JN-DSRCT-1, and BER cell lines [10]. (**A**) Venn diagram depicting overlapping upregulated genes, upon knock-down of *EWSR1-WT1*. (**B**) Venn diagram depicting overlapping downregulated genes, upon knock-down of *EWSR1-WT1*.

The genes that were affected upon knock-down of *EWSR1-WT1* were next compared to a recent study, in which a siRNA-mediated knock-down of the *EWSR1-WT1* fusion gene was performed in the JN-DSRCT-1 and BER cell lines [10]. From the 174 upregulated genes upon *EWS-WT1* knock-down in our OV-054 DSRCT cells, 6% (11/174) showed an overlap with both the JN-DSRCT-1 and BER lines: *CST1*, *FILIP1L*, *CHST1*, *ALCAM*, *EPHA4*, *KRT8*, *ELF3*, *KRT18*, *SLC1A1*, *PCP4,* and *ASS1* (Figure 5A). We found that 9% (7/75) of the downregulated genes, upon *EWSR1-WT1* knock-down, in our OV-054 DSRCT cells were shared with the other two in vitro models: *LOXL3*, *FOXL1*, *RRAD*, *ST6GALNAC5*, *CCL25*, *IGFBP3,* and *C1QL4* (Figure 5B). ShRNA3 resulted in a more efficient knock-down of *EWSR1-WT1*, compared to shRNA 2-mediated knock-down. When only the genes affected upon shRNA 3-mediated knock-down were compared with the JN-DSRCT-1 and BER cell lines, we indeed discovered additional genes to overlap between the three in vitro models (Figure S4). The overlap of the transcriptomic effects, upon knock-down of *EWSR1-WT1* in three independent DSRCT cell lines, confirmed that these genes are direct or indirect targets of the EWSR1-WT1 fusion and potentially play an important role in tumor development and progression.

### 3.4. Drug Screen on OV-054 Cells Reveals Effective Compounds Targeting RTKs and Downstream Pathways

**Figure 6:**
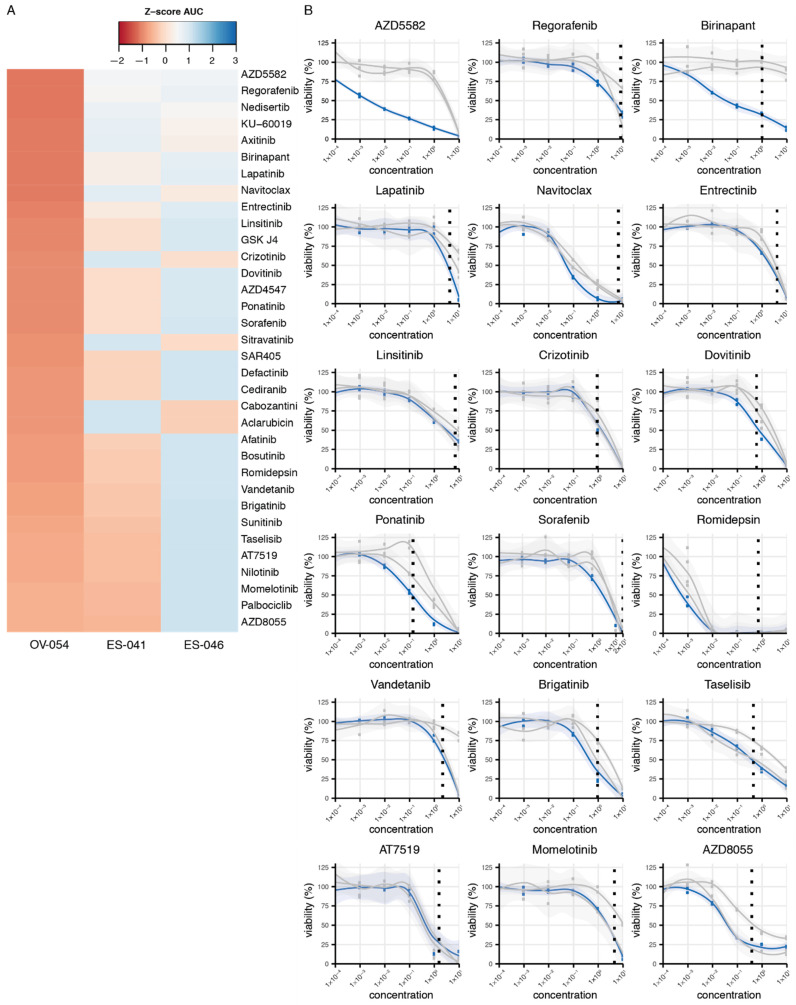
Compound screen on OV-054 DSRCT cells reveals compounds that affect cell viability. (**A**) Heatmap depicting the most sensitive compounds for OV-054 DSRCT, compared to two Ewing sarcoma models (ES-041 and ES-046); colors represent AUC Z-scores. (**B**) Graphs depicting cell viability of OV-054 DSRCT (blue) and the two Ewing sarcoma models (gray), upon a 5-day incubation with different drug concentrations (µM). Known plasma concentrations are shown with the vertical dashed line [23,24,25,26,27,28,29,30,31,32,33].

The established preclinical OV-054 DSRCT in vitro model enabled us to perform a medium-throughput drug screen for DSRCT. The screen involved a panel of 201 different compounds. Several of these compounds affected DSRCT cell viability. The area under the curve (AUC) Z-scores of OV-054 DSRCT cells were compared to the Z-scores of two Ewing sarcoma in vitro models, ES-041 and ES-046, to unravel compounds that showed a higher sensitivity in DSRCT cells (Figure 6A). As DSRCT is closely related to Ewing sarcoma, the compounds showing AUC Z-score possibly present sarcoma specific sensitivity. From these drugs, the compounds were selected that presented with an IC50 value, which was below or around the relevant plasma concentrations (Figure 6B). Several of the compounds that showed an effective response were targeting apoptosis regulators (such as XIAP and BCL2L), including AZD5582, Birinapant, and Navitoclax (Figure 6B), suggesting that the regulation of apoptosis is relevant in DSRCT cells. Interestingly, many other compounds that showed an effective response, including regorafenib, lapatinib, entrectinib, linsitinib, crizotinib, dovitinib, sorafenib, vandetanib, and brigatinib (Figure 6B), target RTKs. Additionally, compounds targeting downstream targets of RTK signaling pathways (Figure 6B), such as the PI3K-AKT and the mTOR signaling pathways, showed effective cell killing, including AT7519 and AZD8055. Together, the drug screening data suggested that the regulation of apoptosis- and RTK-driven signaling pathways are important for DSRCT tumor cell survival.

### 3.5. MERTK, Regulated by EWSR1-WT1, Is a Potential Therapeutic Target in DSRCT

**Figure 7:**
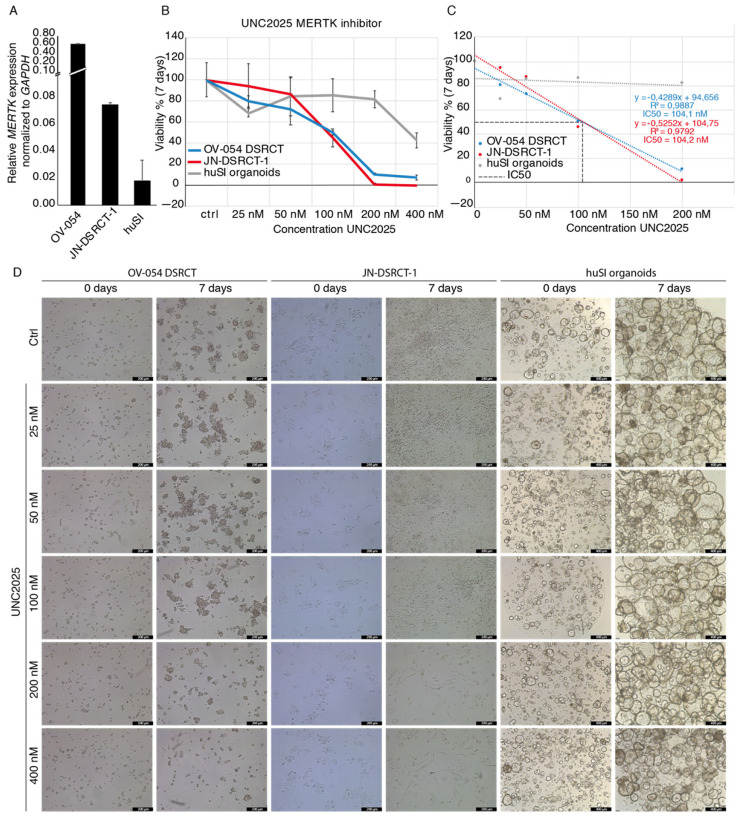
MERTK inhibitor UNC2025 affects DSRCT tumor expansion in vitro. (**A**) Barplot depicting qPCR results of relative *MERTK* expression in OV-054, JN-DSRCT-1, and huSI organoids, normalized to *GAPDH*. (**B**) Graph depicting cell viability of OV-054 DSRCT, JN-DSRCT-1, and huSI organoids, upon administration of 0, 25, 50, 100, 200, and 400 nM UNC2025. (**C**) Graph depicting IC50 of UNC2025 on both OV-054 DSRCT and JN-DSRCT-1 cells (**D**) Representative pictures of OV-054 DSRCT, JN-DSRCT-1, and huSI organoids in vitro, days 0 and 7, after administration of 0, 25, 50, 100, 200, and 400 nM UNC2025 (10× objective).

Since RTKs and downstream pathways of RTKs appeared to be important for DSRCT tumor progression, we looked for RTKs that are regulated by *EWSR1-WT1* expression. *MERTK* is one of the most downregulated genes, upon knock-down of *EWSR1-WT1*, in OV-054 DSRCT cells (Figure 4C) and the JN-DSRCT-1 cell line (Figure 5B), suggesting that high expression of *MERTK* (Figure 7A) is regulated by the EWSR1-WT1 fusion. Interestingly, MERTK has been described as a therapeutic target in several cancers, including melanoma, leukemia, glioblastoma, and gastric cancer [34,35,36]. To investigate the function of MERTK in DSRCT cells, different concentrations of a MERTK/FLT3 small-molecule inhibitor UNC2025 were added to the culture medium, and live cells were counted after 4 and 7 days. Inhibition of MERTK resulted in reduced cell expansion of OV-054 DSRCT cells in vitro in a dose dependent manner (Figure 7B,D), and a similar effect was found in the JN-DSRCT-1 cell line (Figure 7B,D). In both DSRCT cell lines, the IC50 of UNC2025 was around 104 nM (Figure 7C), in line with a previous study of UNC2025-mediated MERTK inhibition in leukemia [34]. Normal human small intestinal (huSI) organoids were exposed to UNC2025 as a control and were only affected by UNC2025 at the highest concentration of 400 nM (Figure 7B,D). This shows that the effect of UNC2025, seen in both DSRCT in vitro models, is a targeted effect, rather than a non-specific cytotoxic effect. Together, this suggested that MERTK is an important driver for cell proliferation in DSRCT and a potential therapeutic target.

## 4. Discussion

DSRCT is a highly aggressive and rare soft tissue sarcoma, characterized by a chromosomal translocation, resulting in the *EWSR1-WT1* gene fusion. Expression of *EWSR1-WT1* presumably regulates the genetic targets responsible for oncogenesis in DSRCT. The 5-year, event-free survival rate is 18% [37], showing the urgence of novel therapies to improve the outcomes for DSRCT patients. 

OV-054 DSRCT cells in vitro grow as nests of small round cells with large nuclei, typically also seen in DSRCT tissue [4]. Both 2D and 3D culture conditions of OV-054 DSRCT consist of a homogeneous cell population, exhibiting similarities to the transcriptional profile of the original tumor. The *EWSR1-WT1* fusion is expressed similarly under 2D and 3D culture conditions. This novel DSRCT in vitro model can be used to investigate molecular pathways driving this rare sarcoma type and further explore therapeutic options, which is urgently needed to improve the poor prognosis of this sarcoma.

To explore the molecular mechanisms of *EWSR1-WT1*, we used an shRNA knock-down approach on our primary DSRCT in vitro model. When the genes that were affected upon shRNA-mediated knock-down of *EWSR1-WT1* were compared with similar gene sets previously described for the JN-DSRCT-1 and BER cell lines [10], we indeed found overlap in up- and downregulated genes. Of note, *ASCL1* was downregulated upon *EWSR1-WT1* knock-down, which was previously described as a direct target of the gene fusion [7]. This confirms that these genes are (in)direct targets of the EWSR1-WT1 fusion and likely play a role in tumor development and progression. 

Gedminas and colleagues show similarities between the molecular mechanisms of EWSR1-FLI1 in Ewing sarcoma and EWSR1-WT1 in DSRCT, despite their different DNA-binding domains [10]. These common features include the dysregulation of the DNA damage response, an alteration in the E2F transcription factor family members and modulation of other pathways, including TGFb and IGF/mTOR signaling. While the mechanism behind these common features is not clear, DSRCT cells show a striking dependence on ERG expression. This is a close family member of FLI1, and it is upregulated by EWSR1-WT1 in DSRCT [10]. Interestingly, Franzetti and colleagues found that a knock-down of *EWSR1-FLI1* expression in Ewing sarcoma affected cell dynamics [38]. Major changes were observed in the dynamics of the actin cytoskeleton and cell-to-cell adhesions shifted to cell-matrix adhesion, associated with an increase of cell migration and invasion potential in vivo. The dynamical changes of the actin cytoskeleton and a shift from cell–cell adhesion to cell-matrix adhesion, shown in the Ewing sarcoma model, are similar to the observations we found in DSRCT, upon knock-down of *EWSR1-WT1* in vitro. In our study, knock-down of *EWSR1-WT1* in DSRCT cells affected cell shape and propagation. We found several cell migration genes and cell–cell adhesion genes to be affected upon *EWSR1-WT1* knock-down. Thus, despite the differences in DNA binding motifs of EWSR1-FLI1 and EWSR1-WT1, the underlying mechanisms driving Ewing sarcoma and DSRCT are possibly similar. 

Because of the rarity of DSRCT, this sarcoma is often excluded from clinical trials. Currently, there are 10 clinical trials ongoing or completed that involve DSRCT patients and just one of these clinical trials included an RTK-targeting compound, i.e., sorafenib (ClinicalTrials.gov identifier: NCT01946529). Unfortunately, the interim analysis of this clinical trial determined that the therapy did not meet the anticipated response; therefore, the trial was stopped. To discover novel therapeutic entities, we performed a medium-throughput drug screen on the established preclinical OV-054 DSRCT in vitro model. A total of 201 different compounds were included in the drug screen for DSRCT cell viability. The compounds to which the OV-054 DSRCT cells were sensitive included several drugs targeting the regulators of apoptosis and many drugs targeting RTKs and downstream pathways of RTKs, including the PI3K-AKT and mTOR signaling pathways. Our data suggests that the regulation of apoptosis is important in DSRCT cells and that RTK-driven pathways are key players in DSRCT tumor progression. Therefore, other compounds that target RTKs or downstream pathways of RTKs might show a more effective response, either alone or in combination with current standard chemotherapy. 

*MERTK* levels in OV-054 DSRCT are decreased upon knock-down of *EWSR1-WT1*, showing that this gene is likely regulated by the fusion protein. Interestingly, *MERTK* expression is higher in the OV-054 DSRCT cells, compared to the JN-DSRCT-1 cells. This inconsistency can be the result of the different fusion breakpoints of the *EWSR1-WT1* fusion genes in the OV-054 DSRCT and JN-DSRCT1 cells. However, we cannot exclude the possibility that this effect is a result of differences in the culture media. Despite these different expression levels of *MERTK* in the different DSRCT in vitro models, the effect of small-molecule inhibitor UNC2025 was similar between the OV-054 DSRCT model and the JN-DSRCT-1 cell line. MERTK has several downstream signaling pathways, including MAPK/ERK, PI3K/AKT, and JAK/STAT, regulating multiple biological processes [35,39,40]. MERTK is involved in multiple malignancies, including leukemia, glioma, melanoma, and rhabdomyosarcoma [35,36], while it has been described as novel therapeutic target in several of these malignancies [36]. Here, we show that MERTK inhibition, with small-molecule inhibitor UNC2025, affects propagation of OV-054 DSRCT cells in vitro, providing a rationale for investigating MERTK as a therapeutic target in DSRCT by small molecule inhibitors, such as UNC2025.

## 5. Conclusions

Using a primary DSRCT patient-derived 2D and 3D cell culture system, we were able to characterize the molecular mechanisms that are driven by the DSRCT-specific EWSR1-WT1 fusion protein. The preclinical DSRCT in vitro model also enables us to perform a medium-throughput drug screen. This screen reveals compounds that affect the cellular pathways that are important for DSRCT cell viability, including RTK-driven signaling pathways. Interestingly, we show that expression the RTK family member *MERTK* correlates with the expression of the *EWSR1-WT1* fusion gene. To our knowledge, this is the first study that reveals effective therapeutic compounds that likely target EWSR1-WT1-driven mechanisms.

## Data Availability

Single-cell RNA sequencing gene counts are provided in Tables S1–S4. RPKM-normalized RNA sequencing data are provided in Tables S6 and S7.

## Supplementary Materials

The following are available online at https://www.mdpi.com/article/10.3390/cancers13236072/s1. Figure S1: effect growth factors on DSRCT cultures, Figure S2: time-lapse DSRCT, upon EWSR1-WT1 knock-down, Figure S3: changes in cell-matrix interactions, Figure S4: comparison target genes to JN-DSRCT-1 and BER cell line, Table S1: scRNAseq_OV-054_2D_TM335.transcripts, Table S2: scRNAseq_OV-054_2D_TM336.transcripts, Table S3: scRNAseq_OV-054_3D_TM93.transcripts, Table S4: scRNAseq_OV-054_3D_TM94.transcripts, Table S5: shRNA sequences, Table S6: RNAseq_EWS-WT1_KD_shR3-NT_rpkm, Table S7: RNAseq_EWS-WT1_KD_shR2-NT_rpkm, Table S8: plasma concentrations compounds.

## References

[1] (2020). Soft Tissue and Bone Tumours: WHO Classification of Tumours.

[2] Bulbul A., Fahy B.N., Xiu J., Rashad S., Mustafa A., Husain H., Hayes-Jordan A. (2017). Desmoplastic small round blue cell tumor: A review of treatment and potential therapeutic genomic alterations. Sarcoma.

[3] Hayes-Jordan A., LaQuaglia M.P., Modak S. (2016). Management of desmoplastic small round cell tumor. Semin. Pediatr. Surg..

[4] Gerald W.L., Rosai J. (1989). Case 2. Desmoplastic small cell tumor with divergent differentiation. Pediatr. Pathol..

[5] Karnieli E., Werner H., Rauscher F.J., Benjamin L.E., LeRoith D. (1996). The IGF-I receptor gene promoter is a molecular target for the Ewing’s sarcoma-Wilms’ tumor 1 fusion protein. J. Biol. Chem..

[6] Gerald W.L., Haber D.A. (2005). The EWS-WT1 gene fusion in desmoplastic small round cell tumor. Semin. Cancer Biol..

[7] Kang H.J., Park J.H., Chen W., Kang S.I., Moroz K., Ladanyi M., Lee S.B. (2014). EWS-WT1 oncoprotein activates neuronal reprogramming factor ASCL1 and promotes neural differentiation. Cancer Res..

[8] Kim J., Lee K., Pelletier J. (1998). The desmoplastic small round cell tumor t(11;22) translocation produces EWS/WT1 isoforms with differing oncogenic properties. Oncogene.

[9] Watson S., Perrin V., Guillemot D., Reynaud S., Coindre J.M., Karanian M., Guinebretiere J.M., Freneaux P., Le Loarer F., Bouvet M. (2018). Transcriptomic definition of molecular subgroups of small round cell sarcomas. J. Pathol..

[10] Gedminas J.M., Chasse M.H., McBrairty M., Beddows I., Kitchen-Goosen S.M., Grohar P.J. (2020). Desmoplastic small round cell tumor is dependent on the EWS-WT1 transcription factor. Oncogenesis.

[11] Markides C.S., Coil D.R., Luong L.H., Mendoza J., Kozielski T., Vardeman D., Giovanella B.C. (2013). Desmoplastic small round cell tumor (DSRCT) xenografts and tissue culture lines: Establishment and initial characterization. Oncol. Lett..

[12] Nishio J., Iwasaki H., Ishiguro M., Ohjimi Y., Fujita C., Yanai F., Nibu K., Mitsudome A., Kaneko Y., Kikuchi M. (2002). Establishment and characterization of a novel human desmoplastic small round cell tumor cell line, JN-DSRCT-1. Lab. Investig..

[13] Hingorani P., Dinu V., Zhang X., Lei H., Shern J.F., Park J., Steel J., Rauf F., Parham D., Gastier-Foster J. (2020). Transcriptome analysis of desmoplastic small round cell tumors identifies actionable therapeutic targets: A report from the Children’s Oncology Group. Sci. Rep..

[14] Bleijs M., van de Wetering M., Clevers H., Drost J. (2019). Xenograft and organoid model systems in cancer research. EMBO J..

[15] Muraro M.J., Dharmadhikari G., Grun D., Groen N., Dielen T., Jansen E., van Gurp L., Engelse M.A., Carlotti F., de Koning E.J. (2016). A single-cell transcriptome atlas of the human pancreas. Cell Syst..

[16] Herman J.S., Sagar, Grun D. (2018). FateID infers cell fate bias in multipotent progenitors from single-cell RNA-seq data. Nat. Methods.

[17] Van Tilburg C.M., Pfaff E., Pajtler K.W., Langenberg K.P.S., Fiesel P., Jones B.C., Balasubramanian G.P., Stark S., Johann P.D., Blattner-Johnson M. (2021). The pediatric precision oncology inform registry: Clinical outcome and benefit for patients with very high-evidence targets. Cancer Discov..

[18] Li H., Durbin R. (2009). Fast and accurate short read alignment with Burrows-Wheeler transform. Bioinformatics.

[19] Da huang W., Sherman B.T., Lempicki R.A. (2009). Bioinformatics enrichment tools: Paths toward the comprehensive functional analysis of large gene lists. Nucleic Acids. Res..

[20] Da Huang W., Sherman B.T., Lempicki R.A. (2009). Systematic and integrative analysis of large gene lists using DAVID bioinformatics resources. Nat. Protoc..

[21] Calandrini C., Schutgens F., Oka R., Margaritis T., Candelli T., Mathijsen L., Ammerlaan C., van Ineveld R.L., Derakhshan S., de Haan S. (2020). An organoid biobank for childhood kidney cancers that captures disease and tissue heterogeneity. Nat. Commun..

[22] Pastushenko I., Blanpain C. (2019). EMT transition states during tumor progression and metastasis. Trends Cell Biol..

[23] Bedi S., Khan S.A., AbuKhader M.M., Alam P., Siddiqui N.A., Husain A. (2018). A comprehensive review on Brigatinib—A wonder drug for targeted cancer therapy in non-small cell lung cancer. Saudi Pharm. J..

[24] Chen E.X., Hotte S., Hirte H., Siu L.L., Lyons J., Squires M., Lovell S., Turner S., McIntosh L., Seymour L. (2014). A Phase I study of cyclin-dependent kinase inhibitor, AT7519, in patients with advanced cancer: NCIC Clinical Trials Group IND 177. Br. J. Cancer.

[25] Juric D., Krop I., Ramanathan R.K., Wilson T.R., Ware J.A., Sanabria Bohorquez S.M., Savage H.M., Sampath D., Salphati L., Lin R.S. (2017). Phase I dose-escalation study of taselisib, an oral PI3K inhibitor, in patients with advanced solid tumors. Cancer Discov..

[26] Kang Y.K., Yoo C., Ryoo B.Y., Lee J.J., Tan E., Park I., Park J.H., Choi Y.J., Jo J., Ryu J.S. (2013). Phase II study of dovitinib in patients with metastatic and/or unresectable gastrointestinal stromal tumours after failure of imatinib and sunitinib. Br. J. Cancer.

[27] Liston D.R., Davis M. (2017). Clinically relevant concentrations of anticancer drugs: A guide for nonclinical studies. Clin. Cancer Res..

[28] Macaulay V.M., Middleton M.R., Eckhardt S.G., Rudin C.M., Juergens R.A., Gedrich R., Gogov S., McCarthy S., Poondru S., Stephens A.W. (2016). Phase I dose-escalation study of linsitinib (OSI-906) and erlotinib in patients with advanced solid tumors. Clin. Cancer Res..

[29] Meneses-Lorente G., Bentley D., Guerini E., Kowalski K., Chow-Maneval E., Yu L., Brink A., Djebli N., Mercier F., Buchheit V. (2021). Characterization of the pharmacokinetics of entrectinib and its active M5 metabolite in healthy volunteers and patients with solid tumors. Investig. New Drugs.

[30] Naing A., Aghajanian C., Raymond E., Olmos D., Schwartz G., Oelmann E., Grinsted L., Burke W., Taylor R., Kaye S. (2012). Safety, tolerability, pharmacokinetics and pharmacodynamics of AZD8055 in advanced solid tumours and lymphoma. Br. J. Cancer.

[31] Wilson W.H., O’Connor O.A., Czuczman M.S., LaCasce A.S., Gerecitano J.F., Leonard J.P., Tulpule A., Dunleavy K., Xiong H., Chiu Y.L. (2010). Navitoclax, a targeted high-affinity inhibitor of BCL-2, in lymphoid malignancies: A phase 1 dose-escalation study of safety, pharmacokinetics, pharmacodynamics, and antitumour activity. Lancet Oncol..

[32] Zheng J., Xin Y., Zhang J., Subramanian R., Murray B.P., Whitney J.A., Warr M.R., Ling J., Moorehead L., Kwan E. (2018). Pharmacokinetics and Disposition of Momelotinib Revealed a Disproportionate Human Metabolite-Resolution for Clinical Development. Drug Metab. Dispos..

[33] Zhu X., Trueman S., Straubinger R.M., Jusko W.J. (2018). Physiologically-based pharmacokinetic and pharmacodynamic models for gemcitabine and birinapant in pancreatic cancer xenografts. J. Pharmacokinet. Pharmacodyn..

[34] DeRyckere D., Lee-Sherick A.B., Huey M.G., Hill A.A., Tyner J.W., Jacobsen K.M., Page L.S., Kirkpatrick G.G., Eryildiz F., Montgomery S.A. (2017). UNC2025, a mertk small-molecule inhibitor, is therapeutically effective alone and in combination with methotrexate in leukemia models. Clin. Cancer Res..

[35] Graham D.K., DeRyckere D., Davies K.D., Earp H.S. (2014). The TAM family: Phosphatidylserine sensing receptor tyrosine kinases gone awry in cancer. Nat. Rev. Cancer.

[36] Schlegel J., Sambade M.J., Sather S., Moschos S.J., Tan A.C., Winges A., DeRyckere D., Carson C.C., Trembath D.G., Tentler J.J. (2013). MERTK receptor tyrosine kinase is a therapeutic target in melanoma. J. Clin. Investig..

[37] Zhang S., Zhang Y., Yu Y.H., Li J. (2015). Results of multimodal treatment for desmoplastic small round cell tumor of the abdomen and pelvis. Int. J. Clin. Exp. Med..

[38] Franzetti G.A., Laud-Duval K., van der Ent W., Brisac A., Irondelle M., Aubert S., Dirksen U., Bouvier C., de Pinieux G., Snaar-Jagalska E. (2017). Cell-to-cell heterogeneity of EWSR1-FLI1 activity determines proliferation/migration choices in Ewing sarcoma cells. Oncogene.

[39] Brandao L.N., Winges A., Christoph S., Sather S., Migdall-Wilson J., Schlegel J., McGranahan A., Gao D., Liang X., Deryckere D. (2013). Inhibition of MerTK increases chemosensitivity and decreases oncogenic potential in T-cell acute lymphoblastic leukemia. Blood Cancer J..

[40] Lee-Sherick A.B., Eisenman K.M., Sather S., McGranahan A., Armistead P.M., McGary C.S., Hunsucker S.A., Schlegel J., Martinson H., Cannon C. (2013). Aberrant Mer receptor tyrosine kinase expression contributes to leukemogenesis in acute myeloid leukemia. Oncogene.

