# Supplementary material for: EWSR1-WT1 Target Genes and Therapeutic Options Identified in a Novel DSRCT In Vitro Model
Source: Cancers (Basel). 2021 Dec 2;13(23):6072. doi: 10.3390/cancers13236072 (PMC8657306; doi:10.3390/cancers13236072)
Supplement: Supplementary file 1 [file cancers-13-06072-s001.zip › Table S5_RNAseq_shRNA sequences EWS-WT1_KD.pdf]

shRNA 2	FW: CCGGCACAACATGCATCAGAGAAACCTCGAGGTTTCTCTGATGCATGTTGTGTTTTT<br>REV: AATTAAAAACACAACATGCATCAGAGAAACCTCGAGGTTTCTCTGATGCATGTTGTG
shRNA 3	FW: CCGGCAGCAGAGTGAGAAACCATACCTCGAGGTATGGTTTCTCACTCTGCTGTTTTT<br>REV: AATTAAAAACAGCAGAGTGAGAAACCATACCTCGAGGTATGGTTTCTCACTCTGCTG
shRNA 4	FW: CCGGATCAGAGAAACATGACCAAACCTCGAGGTTTGGTCATGTTTCTCTGATTTTTT<br>REV: AATTAAAAAATCAGAGAAACATGACCAAACCTCGAGGTTTGGTCATGTTTCTCTGAT
shRNA 5	FW: CCGGGCAGCAGAGTGAGAAACCATACTCGAGTATGGTTTCTCACTCTGCTGCTTTTT<br>REV: AATTAAAAAGCAGCAGAGTGAGAAACCATACTCGAGTATGGTTTCTCACTCTGCTGC
shRNA 6	FW: CCGGTCTCGTTCAGACCAGCTCAAACTCGAGTTTGAGCTGGTCTGAACGAGATTTTT<br>REV: AATTAAAAATCTCGTTCAGACCAGCTCAAACTCGAGTTTGAGCTGGTCTGAACGAGA
shRNA NT	FW: CCGGATCTCGCTTGGGCGAGAGTAAGCTCGAGCTTACTCTCGCCCAAGCGAGATTTTTT<br>REV: AATTAAAAAATCTCGCTTGGGCGAGAGTAAGCTCGAGCTTACTCTCGCCCAAGCGAGAT
