# Supplementary material for: EWSR1-WT1 Target Genes and Therapeutic Options Identified in a Novel DSRCT In Vitro Model
Source: Cancers (Basel). 2021 Dec 2;13(23):6072. doi: 10.3390/cancers13236072 (PMC8657306; doi:10.3390/cancers13236072)
Supplement: Supplementary file 1 [file cancers-13-06072-s001.zip › Table S8_Plasma concentrations.pdf]

**Drug**	**Plasma concentration (μM)**	**Reference**
Regorafenib	8,1	Liston & Davis (2017) (Liston & Davis, 2017)
Birinapant	1,0	Zhu *et al.* (2018) (Zhu *et al*, 2018)
Lapatinib	4,2	Liston & Davis (2017) (Liston & Davis, 2017)
Navitoclax	6,6	Wilson *et al.* (2010) (Wilson *et al*, 2010)
Entrectinib	4,0	Meneses-lorente *et al.* (2021) (Meneses-Lorente *et al*, 2021)
Linsitinib	6,9	Macaulay *et al.* (2017) (Macaulay *et al*, 2017)
Crizotinib	0,9	Liston & Davis (2017) (Liston & Davis, 2017)
Dovitinib	0,6	Kang *et al.* (2013) (Kang *et al*, 2013)
Ponatinib	0,1	Liston & Davis (2017) (Liston & Davis, 2017)
Sorafenib	20,1	Liston & Davis (2017) (Liston & Davis, 2017)
Romidepsin	0,7	Liston & Davis (2017) (Liston & Davis, 2017)
Vandetanib	2,2	Liston & Davis (2017) (Liston & Davis, 2017)
Brigatinib	0,9	Bedi *et al.* (2018) (Bedi *et al*, 2018)
Taselisib	0,4	Juric *et al.* (2017) (Juric *et al*, 2017)
AT7519	1,6	Chen *et al.* (2014) (Chen *et al*, 2014)
Momelotinib	4,5	Zheng *et al.* (2018) (Zheng *et al*, 2018)
AZD8055	0,4	Naing *et al.* (2012) (Naing *et al*, 2012)
