# Supplementary figures and images for: EWSR1-WT1 Target Genes and Therapeutic Options Identified in a Novel DSRCT In Vitro Model
Source: Cancers (Basel). 2021 Dec 2;13(23):6072. doi: 10.3390/cancers13236072 (PMC8657306; doi:10.3390/cancers13236072)

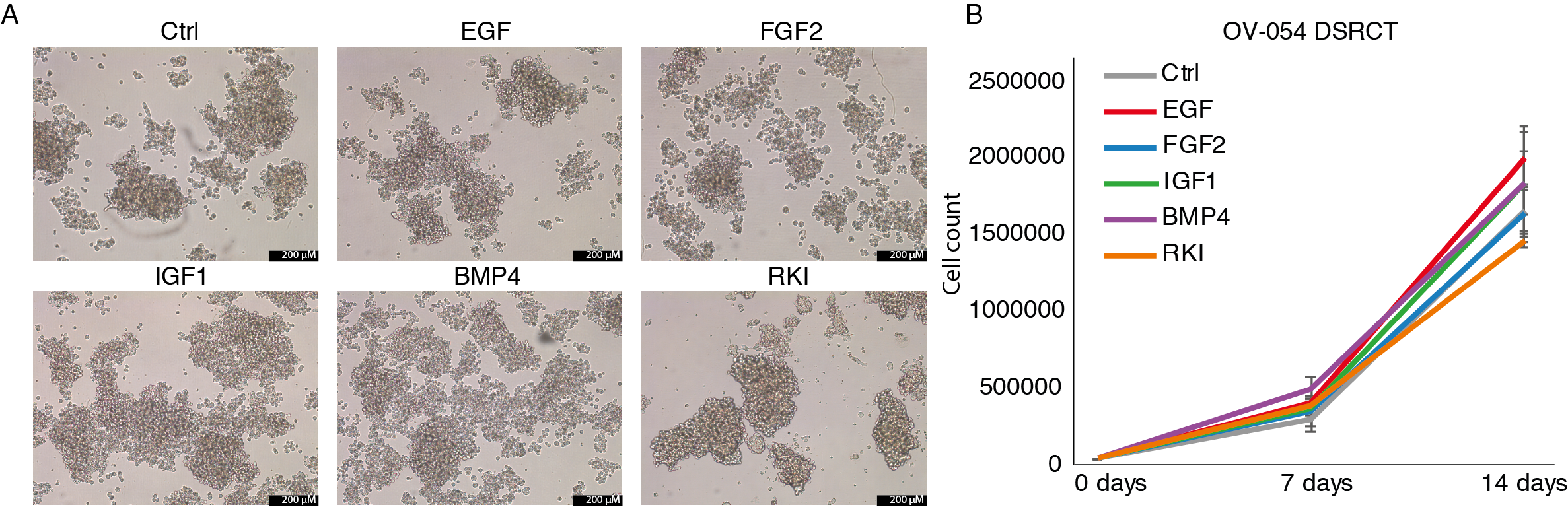

Supplement: Supplementary file 1 [file cancers-13-06072-s001.zip › Fig. s1.png]

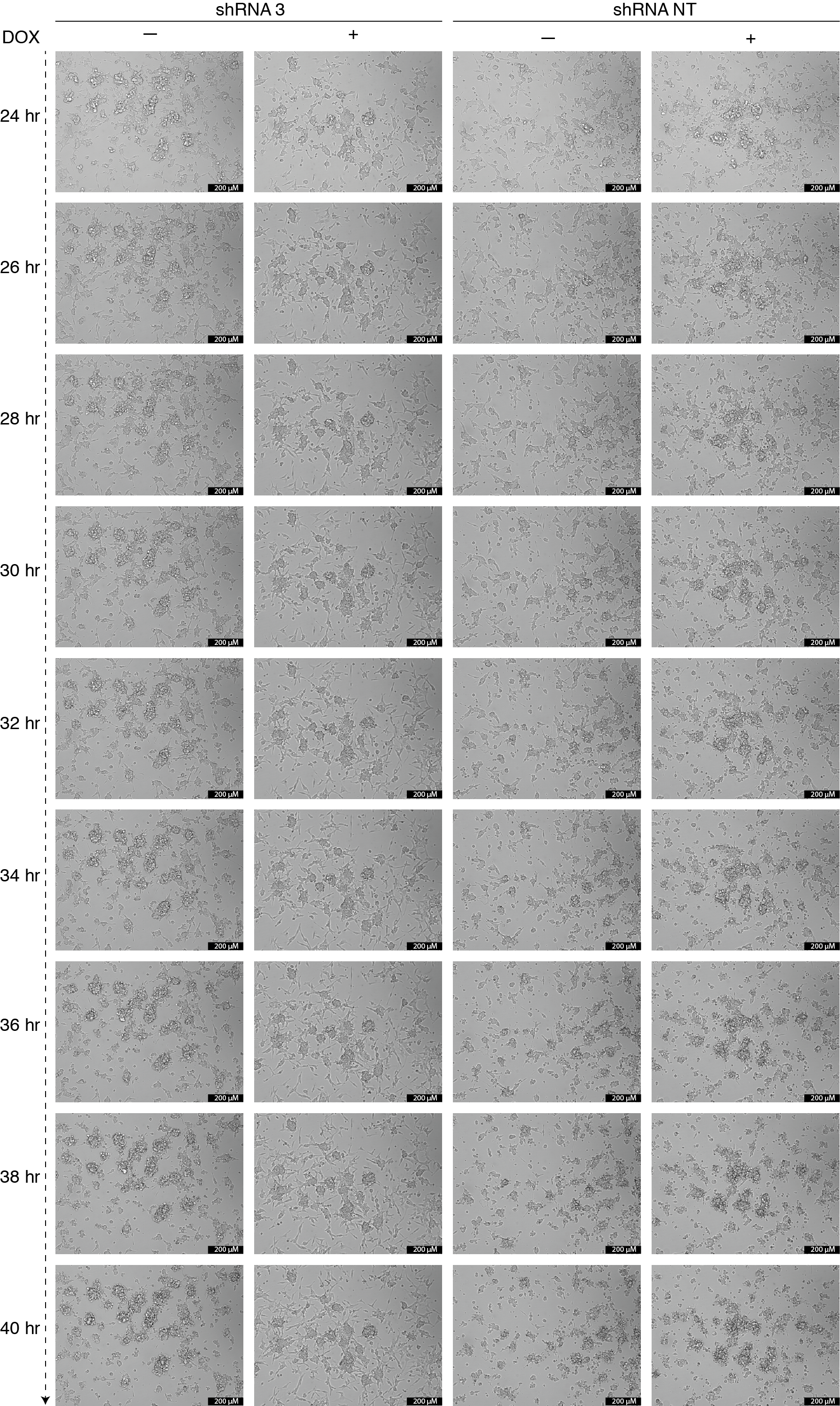

Supplement: Supplementary file 1 [file cancers-13-06072-s001.zip › Fig. s2.png]

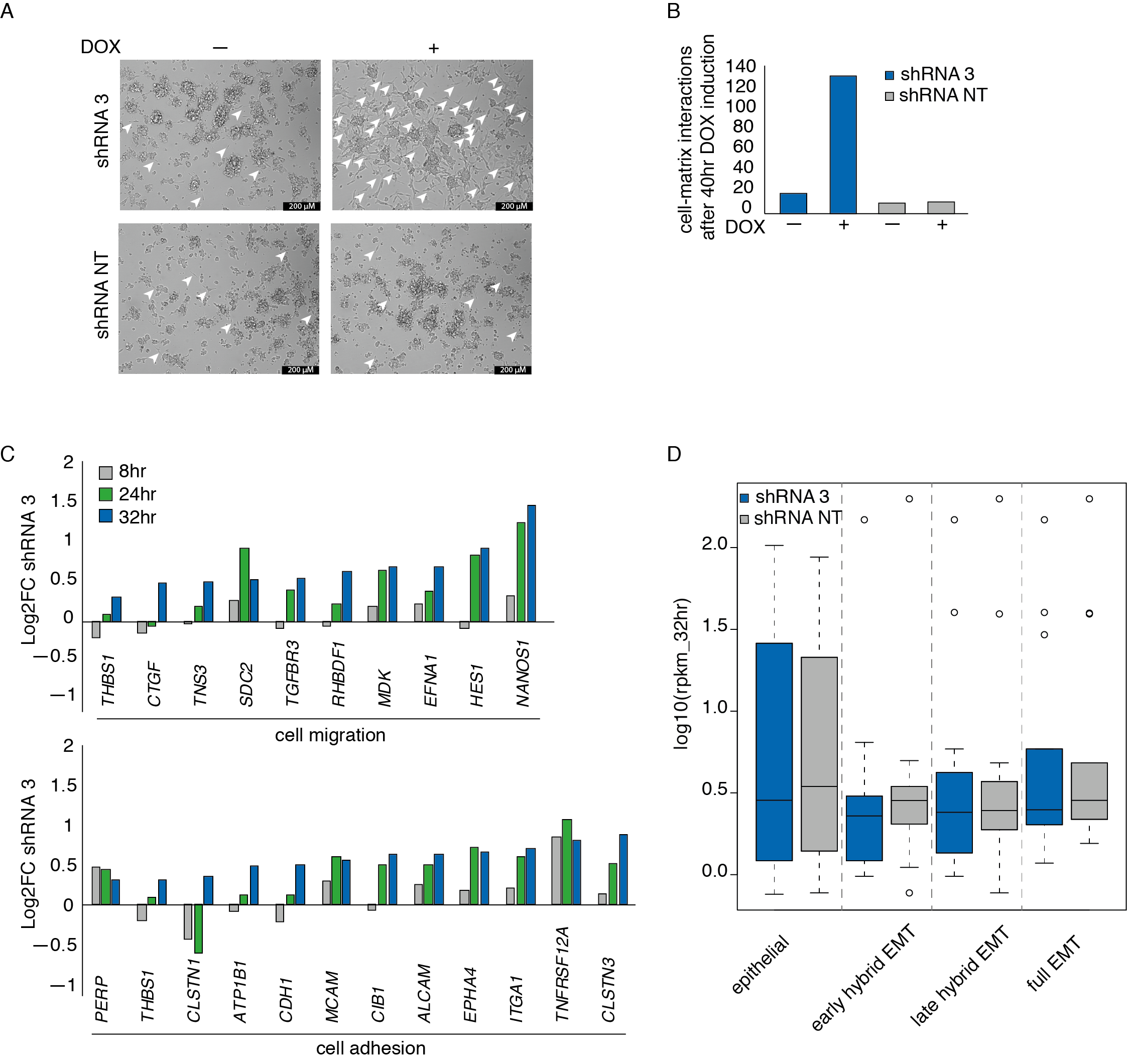

Supplement: Supplementary file 1 [file cancers-13-06072-s001.zip › Fig. s3.png]

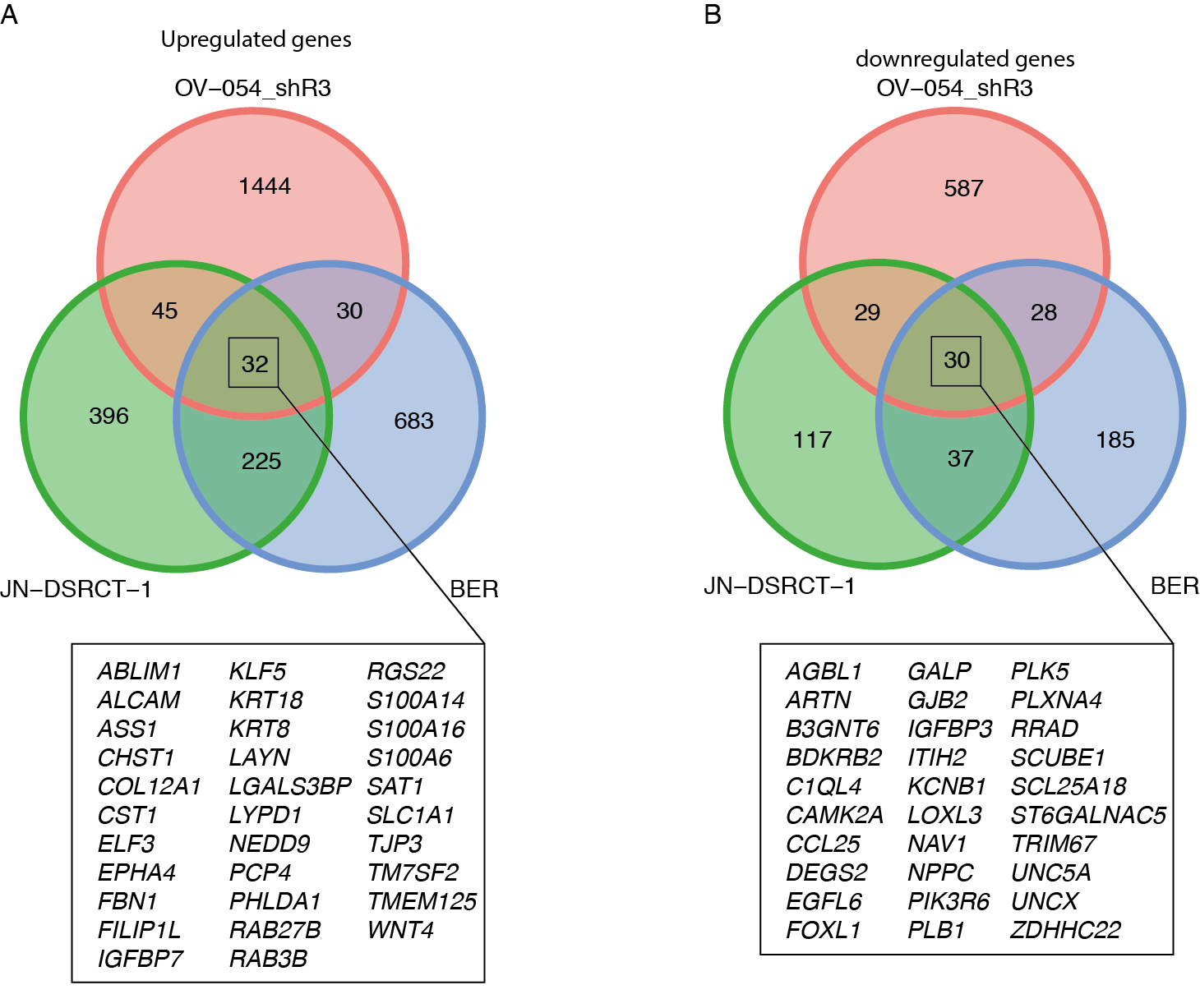

Supplement: Supplementary file 1 [file cancers-13-06072-s001.zip › Fig. s4.png]
